# Autonomous Exploration and Mapping with RFS Occupancy-Grid SLAM

**DOI:** 10.3390/e20060456

**Published:** 2018-06-12

**Authors:** Branko Ristic, Jennifer L. Palmer

**Affiliations:** 1 School of Engineering, RMIT University, Melbourne VIC 3000, Australia; 2 Aerospace Division, Defence Science and Technology Group, Fishermans Bend VIC 3207, Australia

**Keywords:** localisation and mapping, particle filter, Rényi divergence, random finite sets

## Abstract

This short note addresses the problem of autonomous on-line path-panning for exploration and occupancy-grid mapping using a mobile robot. The underlying algorithm for simultaneous localisation and mapping (SLAM) is based on random-finite set (RFS) modelling of ranging sensor measurements, implemented as a Rao-Blackwellised particle filter. Path-planning in general must trade-off between exploration (which reduces the uncertainty in the map) and exploitation (which reduces the uncertainty in the robot pose). In this note we propose a reward function based on the Rényi divergence between the prior and the posterior densities, with RFS modelling of sensor measurements. This approach results in a joint map-pose uncertainty measure without a need to scale and tune their weights.

## 1. Introduction

The task of autonomous exploration and mapping of unknown structured environments combines the solutions to three fundamental problems in mobile robotics: (self)localisation, mapping and motion control. Localisation is the problem of determining the position of the robot within its estimated or a given map. Mapping refers to the problem of integrating robot’s ranging and/or bearing sensor measurements into a coherent representation of the environment. Motion control deals with autonomous decision making (e.g., where to move, where to “look”) for the purpose of accomplishing the mission in the most effective manner (in terms of duration and accuracy).

Algorithms for simultaneous localisation and mapping (SLAM) have been studied extensively in the last decades. While the problem is still an active research area, see [1,2], several state-of-the-art algorithms are already publicly available in the Robot Operating System (ROS) [3,4]. Integrating decision control with SLAM, thereby making the robot autonomous in modelling structured environments, proves to be a much harder problem. Heuristic approaches are reviewed and compared in [5]. More advanced approaches are based on the information gain [6,7,8,9,10].

Autonomous SLAM is a difficult problem because any SLAM algorithm can be seen as an inherently unstable process: unless the robot returns occasionally to already visited and mapped places (this act is referred to as a loop-closure), the pose (position and heading) estimate drifts away from the correct value, resulting in an inaccurate map. Decision making in autonomous SLAM therefore must balance two contradicting objectives, namely exploration and exploitation. Exploration constantly drives the robot towards unexplored areas in order to complete its task as quickly as possible. By exploitation we mean loop-closures: the robot occasionally must return to the already mapped area for the sake of correcting its error in the pose estimate.

Early approaches to robot exploration ignored the uncertainty in the pose estimate and focused on constantly driving the robot towards the nearest frontier, which is the group of cells of the occupancy-grid map on the boundary between the known and unexplored areas [11]. Current state-of-the art in autonomous SLAM is cast in the context of partially-observed Markov Decision processes. The reward function is typically the expected information (defined in the information theoretic framework) resulting from taking a particular action. Entropy reduction in joint estimation of the map and the pose was proposed in a seminal paper [6]. Assuming the uncertainty in pose and the map are independent, the joint entropy can be computed as a sum of two entropies: the entropy of the robot pose and entropy of the map. Several authors subsequently pointed out the drawback of this approach [9,10]: the scale of the numerical values of the two uncertainties is not comparable (the entropy of the map is much higher than the entropy of the pose). A weighted combination of the two entropies was proposed subsequently, with various approaches to balance the two entropies.

Most of the autonomous SLAM algorithms have been developed in the context of a Rao-Blackwellised particle filter (RBPF)-based SLAM. We will remain in this framework and develop an autonomous SLAM for the recently proposed RFS based occupancy-grid SLAM (RFS-OG-SLAM) [2], which is also implemented as a RBPF. In this short note we propose a reward function based on the Rényi divergence between the prior and the posterior joint map-pose densities, with the RFS modelling of sensor measurements. This approach results in a joint map-pose uncertainty measure without a need to scale and tune their respective weights.

## 2. The RFS Occupancy-Grid Based SLAM

The main feature of the RFS-OG-SLAM is that sensor measurements are modelled as a RFS. This model provides a rigorous theoretical framework for imperfect detection (occasionally resulting in false and missed detections) of a ranging sensor. The previous approaches to imperfect detection were based on ad-hoc scan-matching or a design of a likelihood function as a mixture of Gaussian, truncated exponential, and a uniform distribution [12] (Section 6.3).

Let the robot pose be a vector $\theta ={\left[x\;y\;\phi \right]}^{⊺}$ which consists of its position (x,y) in a planar coordinate system and its heading angle ϕ. Robot motion is modelled by a Markov process specified by a (known) transitional density, denoted by $\pi (\theta_{k}|\theta_{k-1},u_{k})$. Here the subscript k∈N refers to time instant $t_{k}$ and $u_{k}$ is the robot-control input applied during the time interval $\tau_{k}=t_{k}-t_{k-1}>0$.

The occupancy-grid map is represented by a vector $m={\left[m_{1}\;m_{2}\;\cdots m_{N}\right]}^{⊺}$, where the binary variable $m_{n}\in \left\{0,1\right\}$ denotes the occupancy of the *n*th grid-cell, n=1,⋯,N, with N≫1 being the number of cells in the grid.

The ranging sensor on the moving robot provides the range (and azimuth) measurements of reflections from the objects within the sensor field of view. Let the measurements provided by the sensor at time $t_{k}$ be represented by a set $Z_{k}=\left\{z_{k,1},\cdots ,z_{k,|Z_{k}|}\right\}$, where $z\in Z_{k}$ is a range-azimuth measurement vector. Both the cardinality of the set $Z_{k}$ and the spatial distribution of its elements are random. For a measurement $z\in Z_{k}$, which is a true return from an occupied grid cell *n* (i.e., with $m_{n}=1$), we assume the likelihood function $g_{n}\left(z|\theta_{k}\right)$ is known. The probability of detecting an object occupying cell *n*, n∈{1,⋯,N} of the map, is state dependent (the probability of detection is typically less than one and may depend on the range to the obstacle, but also other factors, such as the surface characteristics of the object, turbidity of air, a temporary occlusion, etc.) and is denoted as $d_{n}\left(m_{n},\theta_{k}\right)$. Finally, $Z_{k}$ may include false detections modelled as follows: their spatial distribution over the measurement space is denoted by c(z) and their count in each scan is Poisson distributed, with a mean value λ.

The problem is to formulate the Bayes filter, which sequentially computes the joint posterior probability density function (PDF) $p(\theta_{1:k},m|Z_{1:k},u_{1:k})$, where $\theta_{1:k}$ denotes the sequence $\theta_{1},\theta_{2},\cdots ,\theta_{k}$ and likewise $Z_{1:k}\equiv Z_{1},Z_{2},\cdots ,Z_{k}$ and $u_{1:k}\equiv u_{1},\cdots ,u_{k}$.

The solution is formulated using the Rao-Blackwell dimension reduction [13] technique. Application of the chain rule decomposes the the joint posterior PDF as:

(1) $$p\left(\theta_{1:k},m|Z_{1:k},u_{1:k}\right)=p\left(m|Z_{1:k},\theta_{1:k}\right)\;p\left(\theta_{1:k}|Z_{1:k},u_{1:k}\right).$$

Assuming that the occupancy of one cell in the grid-map is independent of the occupancy of other cells (the standard assumption for occupancy-grid SLAM), one can approximate $p(m|Z_{1:k},\theta_{1:k})$ in (1) as a product of $p(m_{n}|Z_{1:k},\theta_{1:k})$ for n=1,⋯,N. Furthermore, the update equation for the probability of occupancy of the *n*-th cell, i.e., $p\left(m_{n}=1|Z_{1:k},\theta_{1:k}\right)=r_{k,n}$, can be expressed analytically [2]:

(2) $$r_{k,n}=\frac{1-\alpha }{1-\alpha \;r_{k-1,n}}\;r_{k-1,n}$$

with

(3) $$\alpha =d_{n}\left(1,\theta_{k}\right)\left[1-\sum_{z\in Z_{k}}\frac{g_{n}\left(z|\theta_{k}\right)}{\lambda \;c(z)}\right].$$

The posterior PDF of the pose, $p(\theta_{1:k}|Z_{1:k},u_{1:k})$ in (1), is propagated using the standard prediction and update equations of the particle filter [13]. However, the likelihood function used in the update of particle weights takes the form $f(Z_{k}|Z_{1:k-1},\theta_{1:k})$ which is equivalent to $f(Z_{k}|r_{k-1},\theta_{1:k})$, with $r_{k}={\left[r_{k,1}\;r_{k,2}\cdots r_{k,N}\right]}^{⊺}$. Assuming the individual beams of the sensor are independent, we can approximate $f(Z_{k}|r_{k-1},\theta_{1:k})$ by a product of likelihoods $p(z|r_{k-1},\theta_{1:k})$ for all $z\in Z_{k}$. Finally,

(4) $$p\left(z|r_{k-1},\theta_{k}\right)\propto \left[1-d_{n_{z}}\left(1,\theta_{k}\right)\;r_{k-1,n_{z}}\right]\lambda \;c\left(z\right)+d_{n_{z}}\left(1,\theta_{k}\right)\;r_{k-1,n_{z}}\;g_{n_{z}}\left(z|\theta_{k}\right)$$

where $n_{z}$ as the nearest grid cell to the point where, for a given pose $\theta_{k}$, range-azimuth measurement z maps to the (x,y) plane.

The RBPF propagates the posterior $p(\theta_{k},m|Z_{1:k},u_{1:k})$, approximated by a weighted set of *S* particles $\{(w_{k}^{\left(i\right)},$ $\theta_{k}^{\left(i\right)},$ $r_{k}^{\left(i\right)}{)\}}_{1\leq i\leq S}$. The weights $w_{k}^{\left(i\right)}$ are non-negative and sum up to one. Each particle consists of a pose sample $\theta_{k}^{\left(i\right)}$ and its associated probability of occupancy vector $r_{k}^{\left(i\right)}$, which represents the estimate of the map.

## 3. Path Planning

Decision making in an autonomous SLAM algorithm includes three steps: the computation of a set of actions; the computation of the reward assigned to each action and selection of the action with the highest reward.

### 3.1. Computing the Set of Actions

**Figure 1:**
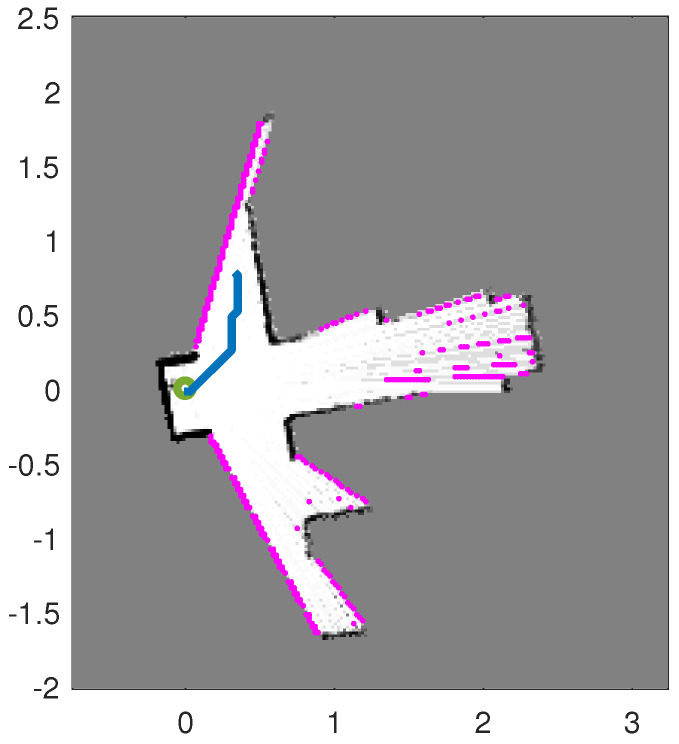
Frontiers and path planing: the image shows a partial map created by the simultaneous localisation and mapping (SLAM) algorithm, using the gray scale to indicate the probability of occupancy (black for probability equal to 1); magenta coloured dots are discovered frontier cells; the robot current position shown as a green circle; a proposed path plotted in blue.

An action is a segment of the total path the robot should follow for the sake of exploring and mapping the environment. The number of actions at a decision time $t_{k}$ (options for robot motion) typically should including both short and long displacements and all possible trajectories to the end points. Due to limited computational resources, in practice only a few actions are typically proposed. They fall into one of the following categories: exploration actions, the place re-visiting (loop-closure) actions, or a combination of the two. The exploration actions are designed to acquire information about unknown areas in order to reduce the uncertainty in the map. Exploration actions are generated by first finding the frontier cells in the map [11,14]. Figure 1 shows a partially built map by a moving robot running the RFS-OG-SLAM algorithm and the discovered frontier cells, using an image processing algorithms based on the Canny edge detector [15]. An exploration action represents a trajectory along the shortest path from the robot’s current pose to the one of the frontiers. Because the number of frontier cells can be large, they are clustered by neighbourhood. The clusters that are too small are removed, while the cells in the centre of the remaining clusters compose a set of exploratory destinations. Subsequently the A* algorithm [16] is applied to find the shortest path from the robot’s current position to each of the exploratory destinations. Because the A* algorithm assumes that the moving robot size equals the grid-cell size, its resulting path can be very close to the walls or the corners. The physical size of the robot is a priori known and therefore the empty space in the map which the robot can traverse is thinned accordingly (using the morphological image processing operations [15]). The place re-visiting actions guide the robot back to the already visited and explored areas.

### 3.2. Reward Function

Reward functions are typically based on a reduction of uncertainty and measured by comparing two different information states. In the Bayesian context, the two information states are the predicted density at time *k* and the updated density at time *k* (after processing the new hypothetical measurements, resulting from the action). However, no measurements are collected before the decision has been made and therefore an expectation operator must be applied with respect to the new measurements resulting from the action.

The reward function can be formulated as the gain defined by an information-theoretic measure, such as the Fisher information, the entropy, the Kullback–Leibler (KL) divergence, etc [17]. We adopt the reward function based on the Rényi divergence between the current and the future information state. The Rényi divergence between two densities, $p_{0}\left(x\right)$ and $p_{1}\left(x\right)$, is defined as [17]:

(5) $$I_{\alpha }\left(p_{1},p_{0}\right)=\frac{1}{\alpha -1}\log\int p_{1}^{\alpha }\left(x\right)p_{0}^{1-\alpha }\left(x\right)dx$$

where α≥0 is a parameter which determines how much we emphasize the tails of two distributions in the metric. In the special cases of α→1 and α=0.5, the Rényi divergence becomes the Kullback–Leibler divergence and the Hellinger affinity, respectively [17].

Using the particle filter approximation of the joint posterior at time k−1, that is ${\left\{\left(w_{k-1}^{\left(i\right)},\theta_{k-1}^{\left(i\right)},r_{k-1}^{\left(i\right)}\right)\right\}}_{1\leq i\leq S}$, the expected reward function for an action $u_{k}$ can be expressed as [18] (Equation 18):

(6) $$R\left(u_{k}\right)\approx \frac{1}{\alpha -1}\sum_{j=1}^{J}\gamma_{1}\left(Z_{k}^{j}|u_{k}\right)\log\frac{\gamma_{\alpha }\left(Z_{k}^{j}|u_{k}\right)}{\gamma_{1}{\left(Z_{k}^{j}|u_{k}\right)}^{\alpha }}$$

where

(7) $$\gamma_{\alpha }\left(Z_{k}|u_{k}\right)=\sum_{i=1}^{S}w_{k-1}^{\left(i\right)}\;{\left[f_{k}\left(Z_{k}|\theta_{k}^{\left(i\right)},r_{k-1}^{\left(i\right)}\right)\right]}^{\alpha },$$

with $\theta_{k}^{\left(i\right)}∼\pi \left(\theta_{k}|\theta_{k-1}^{\left(i\right)},u_{k}\right)$ and $Z_{k}^{j}$ being a draw from $f(Z_{k}|Z_{1:k-1},u_{k})$. Computation of $f_{k}\left(Z_{k}|\theta_{k}^{\left(i\right)},r_{k-1}^{\left(i\right)}\right)$ has been explained at the end of Section 2. Drawing from $f(Z_{k}|Z_{1:k-1},u_{k})$ can be done by ray-casting assuming action $u_{k}$ resulted in pose $\theta_{k}^{\left(i\right)}$ and using the current estimate of the map. In doing so, the probability of a cast ray hitting an object at an occupancy grid cell is made proportional to its probability of occupancy.

An action in the context of active SLAM is a path which can be translated into a sequence of control vectors $u_{k},u_{k+1},\cdots ,u_{k+L}$. Computation of the reward for this case is still given by (6) and (7), but one would have to replace $Z_{k}^{j}$ and $f_{k}\left(Z_{k}|\theta_{k}^{\left(i\right)},r_{k-1}^{\left(i\right)}\right)$ with $Z_{k:k+L}^{j}$ and $f_{k}\left(Z_{k:k+L}|\theta_{k:k+L}^{\left(i\right)},r_{k-1}^{\left(i\right)}\right)$, respectively. This is computationally very demanding and so we approximate that reward as a sum of single step rewards.

The last step of an active SLAM is to select the action with the highest reward and to subsequently execute it. Autonomous SLAM also needs to decide when to terminate its mission. The termination criterion we use is based on the number of frontier cells.

## 4. Numerical Results

**Figure 2:**
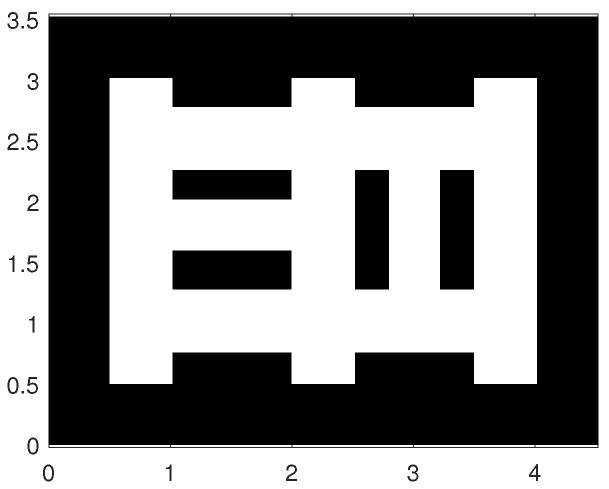
Scenario used in simulations.

Figure 2 shows the map of the area used in simulations for testing and evaluation of the proposed path planning algorithm. The true initial robot pose (distance is measured in arbitrary units, a.u.) is $\left[2.2,\;0.65,\;80^{\circ }\right]$. The SLAM algorithm is initialised with particles of equal weights, zero pose vectors and the probability of occupancy for all cells set to 0.5. The simulated ranging sensor is characterised with a coverage of 360°, angular resolution 0.8°, and $d_{n}\left(1,\theta_{k}\right)=0.8$ if the distance between the robot and *n*-th cell is less than $R_{\max}=2.5$ a.u. False detections are modeled with λ=8 and c(z) as a uniform distribution over the field of view with the radius $R_{\max}$. Standard deviation is set to 0.01 a.u. for range measurements and 0.3° for angular measurements. The occupancy-grid cell size is adopted as 0.02 a.u. The number of particles S=40. The α parameter of the Rényi divergence is set to 0.5. The mission is terminated when none of the clusters of frontier cells has more than 14 members.

**Figure 3:**
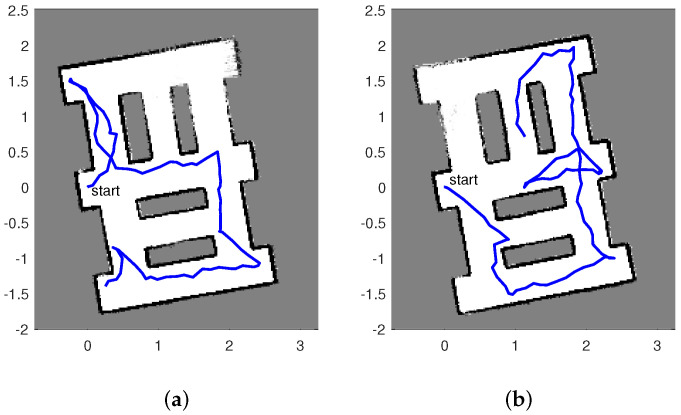
Two maps estimated by the autonomous RFS based occupancy-grid SLAM (RFS-OG-SLAM); blue lines indicate robot trajectories.

Figure 3 displays two estimated maps created by the described autonomous RFS-OG-SLAM for the scenario shown in Figure 2. An estimated map at time *k* is reconstructed from the probability of occupancy vector ${\hat{r}}_{k}$, computed as the average of particles $r_{k}^{\left(i\right)}$, i=1,⋯,S. The maps are tilted because the initial pose of the SLAM algorithm is set to a zero vector. Figure 3 also shows the estimated paths created by the autonomous RFS-OG-SLAM (the blue lines). An estimated path is constructed from the two first components of the mean of the pose particles $\theta_{1:k}^{\left(i\right)}$, i=1,⋯,S. Duration of exploration and mapping is expressed in the number of discrete-time steps required; the maps in Figure 3a,b required 85 and 98 time steps, respectively. The quality of an estimated map at time *k* is expressed by the entropy of vector ${\hat{r}}_{k}$, defined as:

$$H_{k}=-\sum_{n=1}^{N}\left[r_{k,n}\log r_{k,n}+\left(1-r_{k,n}\right)\log\left(1-r_{k,n}\right)\right].$$

Initially, at time k=0, map entropy is $H_{0}=1$. The entropy of the maps in Figure 3a,b are 0.6124 and 0.6074, respectively. An avi movie of a single run of the algorithm can be found in Supplementary Material.

**Figure 4:**
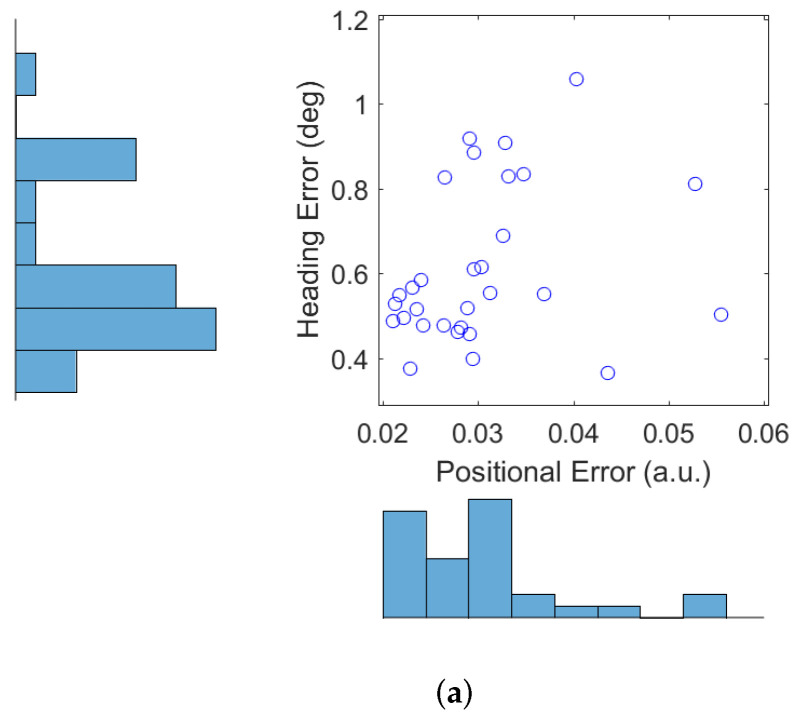

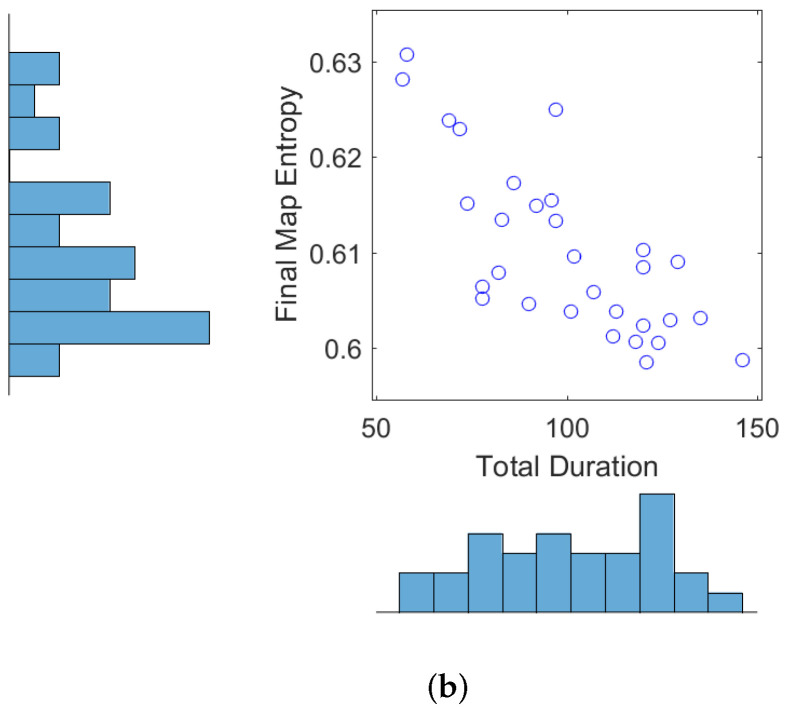
Performance of autonomous RFS-OG-SLAM (from 30 Monte Carlo runs): (**a**) robot position and heading errors; (**b**) final map entropy versus duration of the mission.

**Figure 5:**
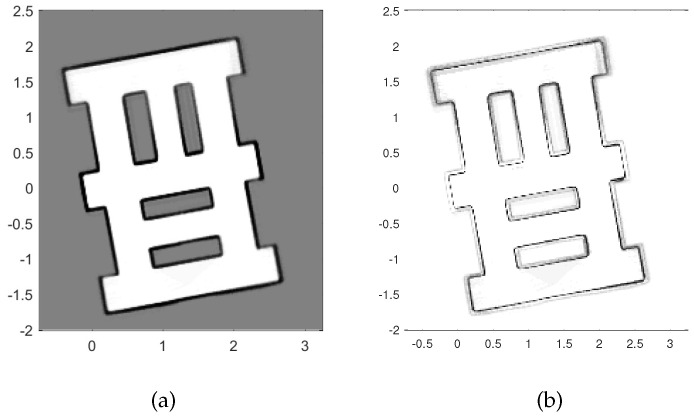
Map statistics from 30 Monte Carlo runs: (**a**) the average estimated map; (**b**) the variance of the estimated map.

Next we show the results obtained from 30 Monte Carlo runs of the autonomous RFS-OG-SLAM, using the simulation setup described above. Figure 4a shows the error of the robot estimated position (in a.u.) and the error of the robot estimated heading (in degrees), averaged over time and over all 30 trajectories. Figure 4b displays the final map entropy versus the duration of the exploration and mapping mission. The average estimated map over 30 Monte Carlo runs is shown in Figure 5a, while the variance is displayed in Figure 5b. Variability in the performance of the autonomous RFS-OG-SLAM is due to many causes, such as the inherent randomness of particle filtering, clustering and reward computation. Overall, however, the proposed autonomous RFS-OG-SLAM performs robustly and produces quality maps without a need for human intervention.

## 5. Conclusions

The note presented a path planning method for autonomous exploration and mapping by a recently proposed RFS occupancy-grid SLAM. The reward function is defined as the Rényi divergence between the prior and the posterior densities, with RFS modelling of sensor measurements. This approach resulted in a joint map-pose uncertainty measure without a need to tune the weighting of the map versus the pose uncertainty. Numerical results indicate reliable performance, combining short exploration with a good quality of estimated maps.

## Supplementary Materials

The supplementary materials are available online at http://www.mdpi.com/1099-4300/20/6/456/s1.

## References

[1] Adams M., Vo B.N., Mahler R., Mullane J. (2014). SLAM gets a PHD: New concepts in map estimation. IEEE Robot. Autom. Mag..

[2] Ristic B., Angley D., Selvaratnam D., Moran B., Palmer J.L. A Random Finite Set Approach to Occupancy-Grid SLAM. Proceedings of the 19th International Conference on Information Fusion.

[3] Santos J.M., Portugal D., Rocha R.P. An evaluation of 2D SLAM techniques available in robot operating system. Proceedings of the 2013 IEEE International Symposium on Safety, Security, and Rescue Robotics (SSRR).

[4] Fang L., Fisher A., Kiss S., Kennedy J., Nagahawatte C., Clothier R., Palmer J. Comparative Evaluation of Time-of-Flight Depth-Imaging Sensors for Mapping and SLAM Applications. Proceedings of the Australian Robotics and Automation Association.

[5] Juliá M., Gil A., Reinoso O. (2012). A comparison of path planning strategies for autonomous exploration and mapping of unknown environments. Auton. Robots.

[6] Stachniss C., Grisetti G., Burgard W. (2005). Information Gain-based Exploration Using Rao—Blackwellized Particle Filters. Robot. Sci. Syst..

[7] Stachniss C., Hähnel D., Burgard W., Grisetti G. (2005). On actively closing loops in grid-based FastSLAM. Adv. Robot..

[8] Blanco L.L., Fernandez-Madrigal J.A., González J. (2008). A novel measure of uncertainty for mobile robot SLAM with Rao-Blackwellized particle filters. Int. J. Robot. Res..

[9] Carlone L., Du J., Ng M.K., Bona B., Indri M. (2014). Active SLAM and exploration with particle filters using Kullback–Leibler divergence. J. Intel. Robot. Syst..

[10] Carrillo H., Dames P., Kumar V., Castellanos J.A. Autonomous robotic exploration using occupancy grid maps and graph SLAM based on Shannon and Rényi entropy. Proceedings of the 2015 IEEE International Conference on Robotics and Automation (ICRA).

[11] Yamauchi B. Frontier-based exploration using multiple robots. Proceedings of the 2nd International Conference on Autonomous Agents.

[12] Thrun S., Burgard W., Fox D. (2005). Probabilistic Robotics.

[13] Doucet A., De Freitas N., Murphy K., Russell S. Rao-Blackwellised particle filtering for dynamic Bayesian networks. Proceedings of the Proceedings of the Sixteenth Conference on Uncertainty in Artificial Intelligence, UAI’00.

[14] Quin P.D., Alempijevic A., Paul G., Liu D. Expanding Wavefront Frontier Detection: An Approach for Efficiently Detecting Frontier Cells. Proceedings of the Australasian Conference on Robotics and Automation.

[15] Gonzalez R.C., Woods R.E., Eddins S.L. (2004). Digital Image Processing Using MATLAB.

[16] Zeng W., Church R.L. (2009). Finding shortest paths on real road networks: The case for A*. Int. J. Geogr. Inf. Sci..

[17] Hero A.O., Kreucher C.M., Blatt D., Hero A.O., Castanòn D., Cochran D., Kastella K. (2008). Information theoretic approaches to sensor management. Foundations and Applications of Sensor Management.

[18] Ristic B., Vo B.N. (2010). Sensor control for multi-object state-space estimation using random finite sets. Automatica.

